# Supplementary material for: Southern Tibetan rifting since late Miocene enabled by basal shear of the underthrusting Indian lithosphere
Source: Nat Commun. 2023 May 4;14:2565. doi: 10.1038/s41467-023-38296-w (PMC10160080; doi:10.1038/s41467-023-38296-w)
Supplement: Supplementary file 10 — Supplementary Data 8 [file 41467_2023_38296_MOESM10_ESM.zip › TP_JIR_05-Mar-2021_14_24_54_SKS_average.pdf]

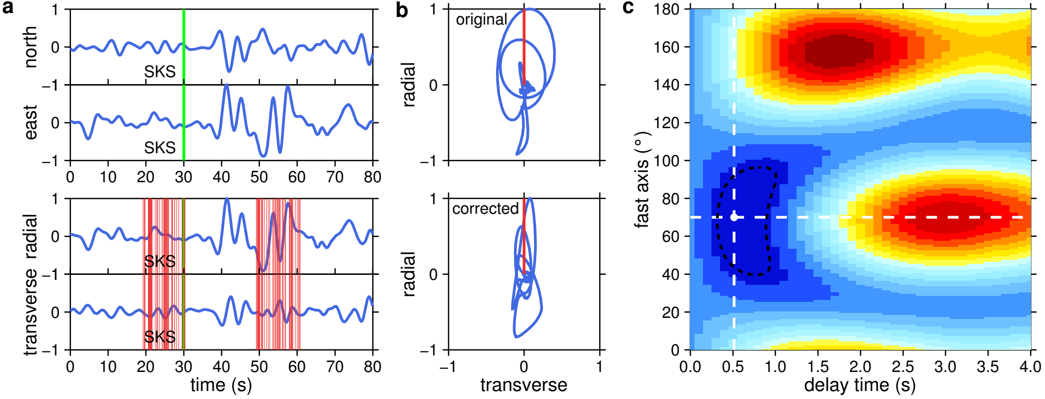
a
north
SKS
east
SKS
radial
SKS
transverse
SKS
time (s)
b
original
radial
corrected
radial
transverse
c
fast axis (°)
delay time (s)
